# Formation of Opioid-Induced Memory and Its Prevention: A Computational Study

**DOI:** 10.3389/fncom.2018.00063

**Published:** 2018-08-02

**Authors:** Mehdi Borjkhani, Fariba Bahrami, Mahyar Janahmadi

**Affiliations:** ^1^CIPCE, Motor Control and Computational Neuroscience Laboratory, School of ECE, College of Engineering, University of Tehran, Tehran, Iran; ^2^Neuroscience Research Center and Department of Physiology, Medical School, Shahid Beheshti University of Medical Sciences, Tehran, Iran

**Keywords:** computational modeling, opioid, memory of addiction, astrocyte, synchronization

## Abstract

There are several experimental studies which suggest opioids consumption forms pathological memories in different brain regions. For example it has been empirically demonstrated that the theta rhythm which appears during chronic opioid consumption is correlated with the addiction memory formation. In this paper, we present a minimal computational model that shows how opioids can change firing patterns of the neurons during acute and chronic opioid consumption and also during withdrawal periods. The model consists of a pre- and post-synaptic neuronal circuits and the astrocyte that monitors the synapses. The output circuitry consists of inhibitory interneurons and excitatory pyramidal neurons. Our simulation results demonstrate that acute opioid consumption induces synchronous patterns in the beta frequency range, while, chronic opioid consumption provokes theta frequency oscillations. This allows us to infer that the theta rhythm appeared during chronic treatment can be an indication of brain engagement in opioid-induced memory formation. Our results also suggest that changing the inputs of the interneurons and the inhibitory neuronal network is not an appropriate method for preventing the formation of pathological memory. However, the same results suggest that prevention of pathological memory formation is possible by manipulating the input of the stimulatory network and the excitatory connections in the neuronal network. They also show that during withdrawal periods, firing rate is reduced and random fluctuations are generated in the modeled neural network. The random fluctuations disappear and synchronized patterns emerge when the activities of the astrocytic transporters are decreased. These results suggest that formation of the synchronized activities can be correlated with the relapse. Our model also predicts that reduction in gliotransmitter release can eliminate the synchrony and thereby it can reduce the likelihood of the relapse occurrence.

## Introduction

Addiction is believed to be a pathological memory which is formed in different brain regions, especially in the Ventral Tegmental Area (VTA), Nucleus Accumbens (NA), Prefrontal Cortex (PC), and the hippocampus (Berke and Hyman, 2000; Boening, 2001; Nestler, 2001, 2013; Kauer and Malenka, 2007; Kalivas and O'Brien, 2008; Dong and Nestler, 2014). Drugs generate the pathological memories by affecting the glutamatergic and dopaminergic systems in the neural circuitry of the brain (Capogna et al., 1993; Contet et al., 2004; Peters and De Vries, 2012; Chartoff and Connery, 2014; García-Pérez et al., 2015; Wang et al., 2017). It has been suggested that such pathological memories are responsible for relapse during the withdrawal periods (Crombag et al., 2008; Vengeliene et al., 2015). Since the opioids are diversely used in treatments, their addictive properties require further attention. It has been shown that activation of the opioid receptors can decrease the release of the GABA with a disinhibitory mechanism, and as a result glutamatergic transmissions increase (Caudle and Chavkin, 1990; Chen and Marine, 1991; Capogna et al., 1993; Kow et al., 2002; McQuiston and Saggau, 2003; Blaesse et al., 2015). These modifications can contribute to memory formation process (Borjkhani et al., 2018). Furthermore, many researchers suggested that the astrocytes play fundamental roles in the formation of addiction (López-Hidalgo et al., 2012; Borjkhani et al., 2014, 2018; Scofield and Kalivas, 2014; Harada et al., 2015; Wang et al., 2017).

Among the wide disciplines of researches on addiction, simulation studies use computational tools to developed computational models driven from experimental observations. Simulation of the computational models have the potential to predict new aspects of this process (Borjkhani et al., 2018). One of the computational models for neurons that has received huge attentions is the model introduced by Izhikevich for the mammalian neurons (Izhikevich, 2003). This model has been widely examined and used in various computational neuroscience studies (Reato et al., 2012; Thibeault and Srinivasa, 2013; Nobukawa et al., 2015a,b, 2017; Seyed-Allaei, 2015; Nobukawa and Nishimura, 2016; Sahasranamam et al., 2016; Kondo et al., 2017). Although this model has a simple structure, it can reproduce most of the major firing patterns of different neurons (Izhikevich, 2004; Nobukawa et al., 2017). In this paper, we used this model to describe the behavior of the post-synaptic neurons in the neural circuitry. The model of the neural circuitry is enhanced by adding the function of the astrocyte to it. This model is used to examine firing patterns of neurons in various addiction modes under the influence of opioids.

It has been empirically shown that synchronous firing activities in the neural circuitry are correlated with the formation of memory (Axmacher et al., 2006; Fell and Axmacher, 2011). In our simulations, synchronized fluctuations were observed in both chronic and withdrawal modes. Emergence of the synchronized firing activities during chronic opioid consumption can be linked to the memory formation process, and their appearance during withdrawal mode is most probably an indication of a memory recall that has already been formed.

## Materials and methods

### Modeling approach

**Figure 1:**
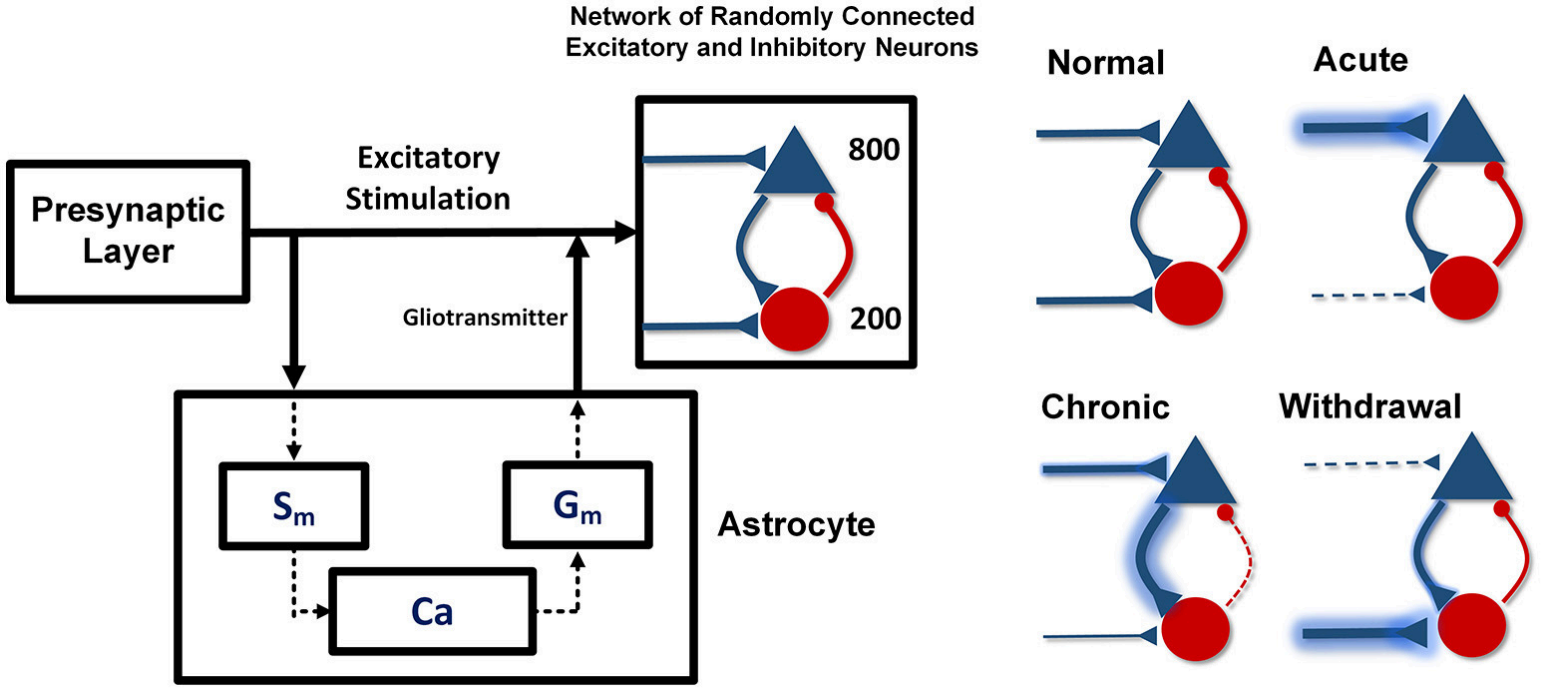
The proposed network model. Pyramid neurons are shown with the triangles and the interneurons with the circles. The pre-synaptic layer produces a random signal that stimulates the astrocyte and the post-synaptic network. The astrocyte also stimulates the post-synaptic network by releasing gliotransmitter. In the acute state of addiction, the inputs of the excitatory neurons are larger and the inputs of the inhibitory neurons are smaller than the stimulation amplitude in the normal case. In the chronic mode, the inputs of the excitatory neurons are slightly smaller and the inputs of the inhibitory neurons are slightly larger than the input amplitude in the acute state. In the withdrawal state, the inputs of the stimulatory neurons decrease significantly and the inputs of the inhibitory neurons are considerably larger than the inputs of the normal state.

The proposed model includes the pre- and post-synaptic layers and one astrocyte (Figure 1). The pre-synaptic layer generates a random output signal with a normal distribution which is received by the post-synaptic network and the astrocyte. Stimulation of the astrocyte induces calcium oscillations in it, which leads to the gliotransmitter release. The released gliotransmitter, along with the stimulatory signal of the pre-synaptic layer, affect the post-synaptic layer. In the presented model, the effect of astrocytic transporters is defined by a parameter that modulates the strength of the stimulatory signals.

Acute consumption of opioids, using the disinhibitory mechanism, enhances the input of the excitatory neurons and reduces the input of the interneurons (Chartoff and Connery, 2014). In this case, synaptic connections between the neurons have their normal weights. If uptake of the substance continues and the chronic condition is developed, in addition to the changes in the input of the neurons, synaptic connections between the neurons will also be modified. As a result, the excitatory connections become stronger and the inhibitory connections in the network become weaker. In chronic consumption of the opioids, despite the fact that the inputs of the excitatory neurons are larger and the inputs of the interneurons are smaller than in the normal state (Gähwiler, 1980; Chen and Marine, 1991; Chen and Huang, 1992; Akaishi et al., 2000), however, due to the tolerance properties of opiates, stimulatory signals tend to return to the normal state. Also, long-term use of the opioids can strengthen the excitatory synapses and weaken the inhibitory ones. Strengthening of the excitatory connections is due to the phosphorylation of the CaMKII and AMPARs, which leads to LTP induction (Chen and Marine, 1991; Wolf, 2003; Kauer and Malenka, 2007; van Huijstee and Mansvelder, 2015). Besides that, attenuation of the inhibitory synaptic connections is due to LTD induction in the inhibitory synapses (Cohen et al., 1992; Capogna et al., 1993; McQuiston and Saggau, 2003; Zachariou et al., 2013; Blaesse et al., 2015).

In the withdrawal period, due to the lack of opioids and the absence of the disinhibitory mechanism, the inputs of the excitatory neurons are weaker, and the inputs of the inhibitory ones are stronger than in the normal state (Chartoff and Connery, 2014). Furthermore, over time, synaptic connections in the neuronal network are going to attain a normal state.

### Post-synaptic network model

We modeled the post-synaptic network using Izhikevich's spiking model for mammalian cortex (Izhikevich, 2003). It has been assumed that 1,000 neurons are randomly connected to each other. The ratio of the excitatory to the inhibitory neurons is 4 to 1 (800 neurons are excitatory, and 200 neurons are inhibitory). Synaptic connections have been represented by *S* = [*s*_*Exc*_*g*_1_, *s*_*Inh*_*g*_2_]. Here, *g*_1_ is a 1000^*^800 matrix with random entries which represent the strength of the connections in the excitatory network, and *g*_2_ is a 1000^*^200 matrix with random entries which denotes the inhibitory network connections. *s*_*Exc*_ = 0.5 and *s*_*Inh*_ = −1 are constants that show the strength of the excitatory and the inhibitory connections. The *ith* neuron of the network is described by (Izhikevich, 2003):

$$\begin{array}{rcl} v_{i}^{\prime } & = & 0.04v_{i}^{2}+5v_{i}+140-u_{i}+I_{Exc/Inh}+I_{Astro} \end{array}$$

$$\begin{array}{rcl} u_{i}^{\prime } & = & a_{i}\left(b_{i}v_{i}-u_{i}\right) \end{array}$$

(1) $$if\;v_{i}\geq 30\;mv,\;then\{\begin{array}{c} v_{i}\;\leftarrow c\;\\ u_{i}\;\leftarrow u_{i}+d \end{array}$$

Here *v*_*i*_ and *u*_*i*_ are membrane voltage and the recovery variable of the *ith* neuron in the network. Parameters of the excitatory neurons are (*a*_*i*_, *b*_*i*_) = (0.02, 0.2) and $\left(c_{i},d_{i}\right)=\left(-65,8\right)+\left(15,-6\right){\text{r}}_{i}^{2}$. Parameters of the inhibitory neurons are (*a*_*i*_, *b*_*i*_) = (0.02, 0.25) + (0.08, −0.05)r_*i*_, and (*c*_*i*_, *d*_*i*_) = (−65, 2). Here, *r*_*i*_ represents a random variable with a uniform distribution between [0,1]. *I*_*Astro*_ denotes the astrocyte-mediated current. The input of the excitatory (*I*_*Exc*_) and the inhibitory (*I*_*Inh*_) networks are defined as follow:

(2) $$\begin{array}{r} I_{Exc}=\delta K_{Exc}f_{1}+I_{Syn} \end{array}$$

(3) $$\begin{array}{r} I_{Inh}=\delta K_{Inh}f_{2}+I_{Syn} \end{array}$$

Where, *f*_1_ and *f*_2_ are normally distributed random numbers (with zero mean and σ = 1). *K*_*Exc*_ = 5 and *K*_*Inh*_ = 2 are the amplitude of the excitatory and the inhibitory applied currents. *I*_*syn*_ =_*fired*_ represents synaptic input from the network; Fired neurons can influence synaptic input with this parameter. The parameter δ = 0.8 has been added to the model and denotes a constant that relates to the activity of astrocyte transporters. When the activity of the astrocyte transporters is high, δ = 0.5 and when the activity is zero, the parameter is considered to be δ = 1.

### Astrocyte model

The model used to describe the astrocyte-neuron interaction was developed originally by Postnov and colleagues (Postnov et al., 2007, 2009). In this model, the signal received by astrocyte causes calcium fluctuations. Calcium oscillations formed in the astrocyte lead to gliotransmitter release. The model is described by the following set of equations:

(4) $$\begin{array}{rcl} 0.05*\frac{dc}{dt} & = & -c-\left(2/0.04\right)f\left(c,c_{e}\right)+\left(0.31+0.006*S_{m}\right) \end{array}$$

(5) $$\begin{array}{rcl} 0.04 & * & 0.05*\frac{dc_{e}}{dt}=f\left(c,c_{e}\right) \end{array}$$

(6) $$\begin{array}{rcl} f\left(c,c_{e}\right) & = & 0.13*\frac{c^{2}}{1+c^{2}}-\left(\frac{c_{e}^{2}}{1+c_{e}^{2}}\right)\left(\frac{c^{4}}{0.9^{4}+c^{4}}\right) \end{array}$$

$$\begin{array}{r} -0.004c_{e} \end{array}$$

$$\begin{array}{rcl} 5*\frac{dS_{m}}{dt} & = & \left(1+\tanh\left[5*\left(K_{Exc}|f_{1}|-0.45\right)\right]\right)\times \left(1-S_{m}\right) \end{array}$$

(7) $$\begin{array}{r} -\frac{S_{m}}{3} \end{array}$$

(8) $$\begin{array}{rcl} 0.025*\frac{dG_{m}}{dt} & = & \left(1+\tanh\left[10*\left(c-0.5\right)\right]\right)\times \left(1-G_{m}\right)-\frac{G_{m}}{3} \end{array}$$

(9) $$\begin{array}{rcl} I_{Astro} & = & K_{Glio}\delta G_{m} \end{array}$$

Where *c* and *c*_*e*_ are the concentrations of calcium in the cytoplasm of the astrocyte and inside the endoplasmic reticulum, respectively. The second messenger *S*_*m*_(*IP*_3_) modulates the calcium influx from the extracellular space. *f*(*c, c*_*e*_) which is a nonlinear function, defines the calcium exchange between the cytoplasm and the endoplasmic reticulum. *G*_*m*_ defines the gliotransmitter dynamics and depends on the astrocyte's calcium concentration. *K*_*Glio*_ is a constant which controls the gliotransmitter release.

## Results

**Figure 2:**
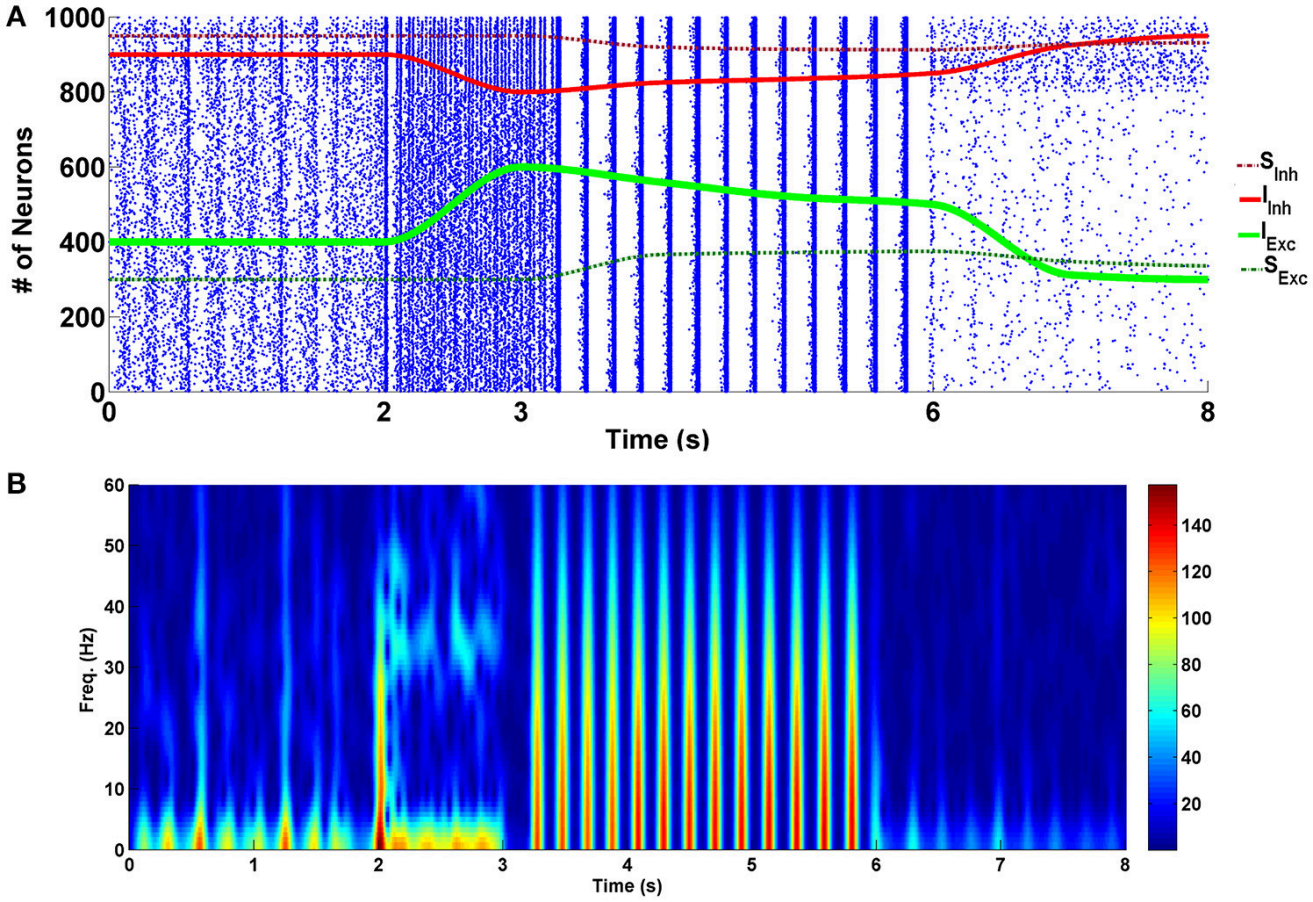
Simulation results for four different modes of the addiction-memory formation process. **(A)** The raster plot of neural activities in the post-synaptic network. Changes in the strength of the inhibitory synaptic connections are shown on the same plot with the red dashed line, strength of the input of interneurons with the red line, strength of the input of excitatory neurons with the green line, and the strength of the excitatory synaptic connections with the dashed green line. **(B)** The spectrogram of the firing neurons. The graph from zero to 2 s corresponds to the normal mode, the acute mode is shown from 2 to 3 s, the chronic state is from 3 to 6 s, and the withdrawal mode is from 6 to 8 s.

The simulation results of the four different modes are presented in Figure 2. Raster plot of fired neurons has been illustrated in the top panel. The bottom panel describes the spectrogram of the population spike count (PSC). Power of the frequency components over time can be observed there. Between 0 and 2 s, the network is in the normal state. In the normal conditions default values for the parameters—which have been described in the modeling approach part—are used in the simulations. Between moments of 2 to 3 s, the input of excitatory neurons (shown with the green line) is increased by 50% (*K*_*Exc*_ = 7.5), and the input of the inhibitory neurons (indicated by the red line) is decreased by 50% (*K*_*Inh*_ = 1). This range indicates the acute state of drug intake. It can be seen that some of the neurons in the network are going to synchronize. Spectrogram of PSCs denotes that power of the frequency components in fired neurons in less than 10 and 40 Hz is dominant.

During the time between 3 and 6 s, we continuously changed the inputs of excitatory and inhibitory neurons to get closer to the normal state. At the same time, excitatory connections (shown with green dashed line) in the network were strengthened, and inhibitory connections (demonstrated with red dashed line) were weakened manually. This range reflects the chronic form of drug use. Changes in synaptic weights reflect the long-term effects of drug use. Here, it is assumed that the amplitude of the excitatory neuron's input is enhanced by 25% (*K*_*Exc*_ = 6.25) in equation (2) and the amplitude of the interneurons input is diminished by 25% (*K*_*Inh*_ = 1.5) in equation (3). Besides that, the strength of the excitatory synaptic connections is increased by 25% and inhibitory synaptic connections is decreased by 25% compared to the normal mode (*s*_*Exc*_ = 0.625, and *s*_*Inh*_ = −0.75). Based on the results, it can be seen that neurons are synchronized in the theta frequency range (5–10) Hz. Spectrogram of the firing neurons over time demonstrates that power of the theta band frequencies is higher than the normal level. Furthermore, raster plot indicates that neurons fire with the burst patterns.

The withdrawal mode is shown between 6 and 8 s. In this case, the input of excitatory neurons tends to be lower than normal (*K*_*Exc*_ = 3.75), and the input of inhibitory neurons tends to be more than normal mode (*K*_*Inh*_ = 2.5). Since in withdrawal period, connections in the network of the neurons are going to approach the normal state, synaptic strengths have been changed to *s*_*Exc*_ = 0.56, and *s*_*Inh*_ = −0.875. It can be seen that produced spikes in the network are irregular and no synchronous patterns can be observed here.

**Figure 3:**
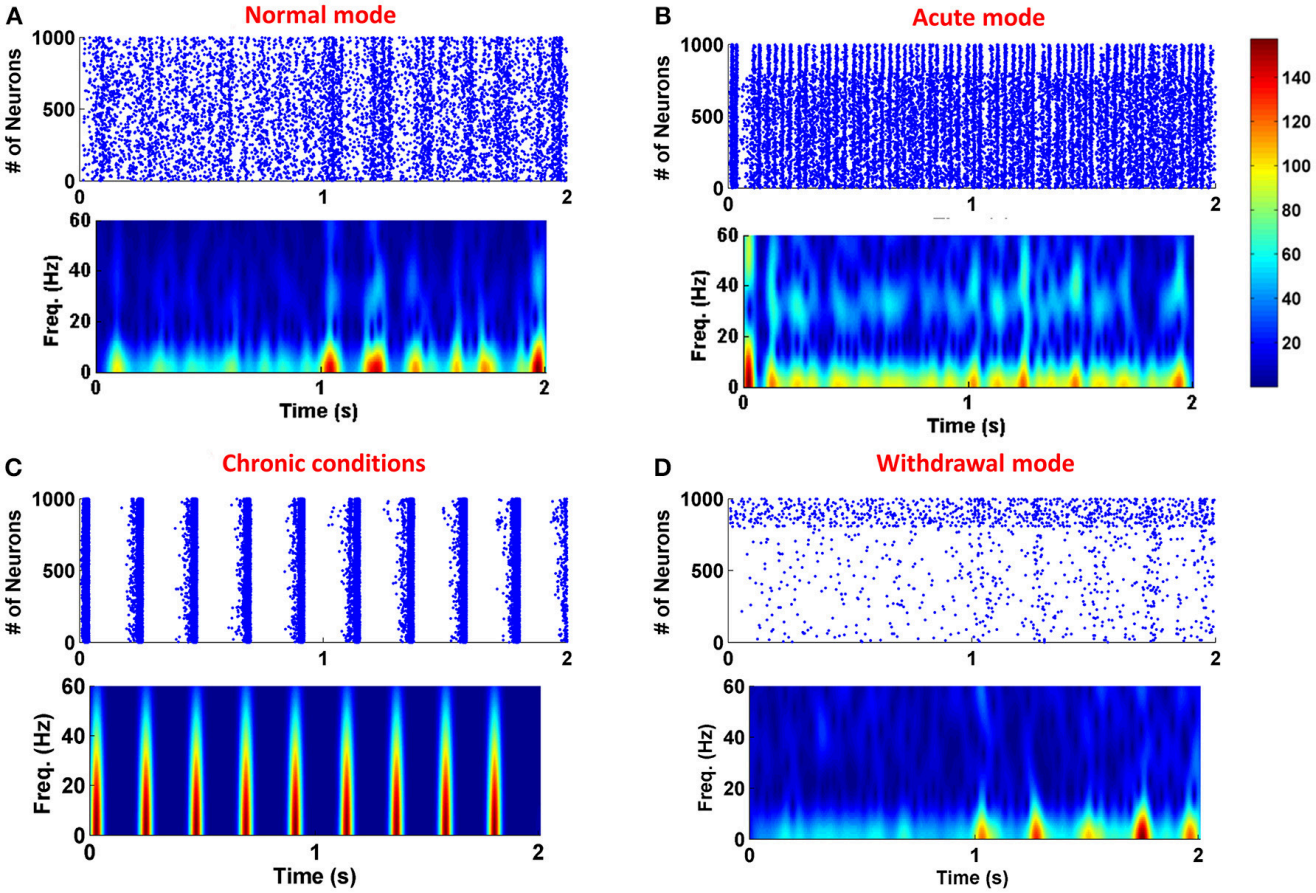
Simulation results for four different modes of the addiction formation process without (before 1 s) and with (from 1 to 2 s) astrocytic gliotransmitter release. **(A)** Normal mode, **(B)** Acute consumption, **(C)** Chronic consumption, and **(D)** Withdrawal mode. In each simulation from zero to 1 s, astrocyte does not release any gliotransmitter, and from 1 to 2 s it releases gliotransmitter. In each panel, top figure represents the raster plot and the bottom one shows the spectrogram of PSCs.

To show the role of astrocytic gliotransmitter release in various forms of addiction, simulations have been performed with and without the presence of the astrocyte in the neuronal network. Figure 3 shows the results of simulations; panel (A) shows the normal mode, panel (B) demonstrates the acute state, panel (C) denotes the chronic consumption conditions, and panel (D) illustrates the withdrawal mode. In all simulations, it is assumed that the gliotransmitter is not released before 1 s, and between 1 and 2 s, the astrocytic gliotransmitter is released. The simulation results indicate that during normal conditions, gliotransmitter release causes low-frequency synchronized oscillations. In the case of acute drug consumption, the release of gliotransmitter leads to an increase in low-frequency synchronous oscillations. As shown in Figure 3C, the release of astrocytic gliotransmitter does not affect the formation of synchronous oscillations during chronic drug consumption. Therefore, it can be concluded that the formation of pathological memory is more due to the change in the neurotransmission and not the gliotransmission. In the withdrawal period shown in Figure 3D, it is observed that the release of the gliotransmitter causes the formation of low-frequency synchronous oscillations.

## Discussion

Opioids like other drugs of abuse can manipulate neural functions and structures (Hyman et al., 2006; Kauer and Malenka, 2007). This manipulation may lead to formation of new memories related to addiction (Nestler, 2001, 2002, 2013; Dong and Nestler, 2014). In fact, one can call drug-induced memories “the memory of addiction”. The formation of such memories may contribute to relapse in withdrawal period (Boening, 2001). Most of the researchers believe that in the acute opioid consumption, the activities of the inhibitory neurons diminishes and the activity of the excitatory neurons increases (Williams et al., 2001; McQuiston and Saggau, 2003; Chartoff and Connery, 2014). Inspired by those empirical researches, in the proposed model, we increased the inputs of the excitatory neurons and decreased the inputs of the interneurons. In this case, the simulation results indicated that about 5% of the neurons exhibited synchronized firing patterns.

On the other hand, repetitive usage of the opioids can induce long-term effects on the neurons through induction of LTP and LTD in the excitatory and inhibitory synapses, respectively (Lou et al., 1999; Heidari et al., 2013). Therefore, over time, excitatory synapses are strengthened and inhibitory synapses are weakened. Our simulation results show that in this situation, almost all of the neurons have been synchronized in the theta frequency range (5–10 Hz). Here, introduction of the theta rhythm in the PSC of the neural circuitry can be meaningful. In other words, in the experiments it has been observed that theta rhythm is involved in learning and memory formation process in rodents, especially in the hippocampal neurons (Vertes, 2005). Consequently, we can interpret the appearance of the theta band frequencies in our simulations as drug-related memory formation. Furthermore, Przewlocki et al. reported that in the chronic opioid consumption, burst patterns are observed empirically in the affected neurons (Przewlocki et al., 1999), this was exactly what our model predicted too (Figure 2).

**Figure 4:**
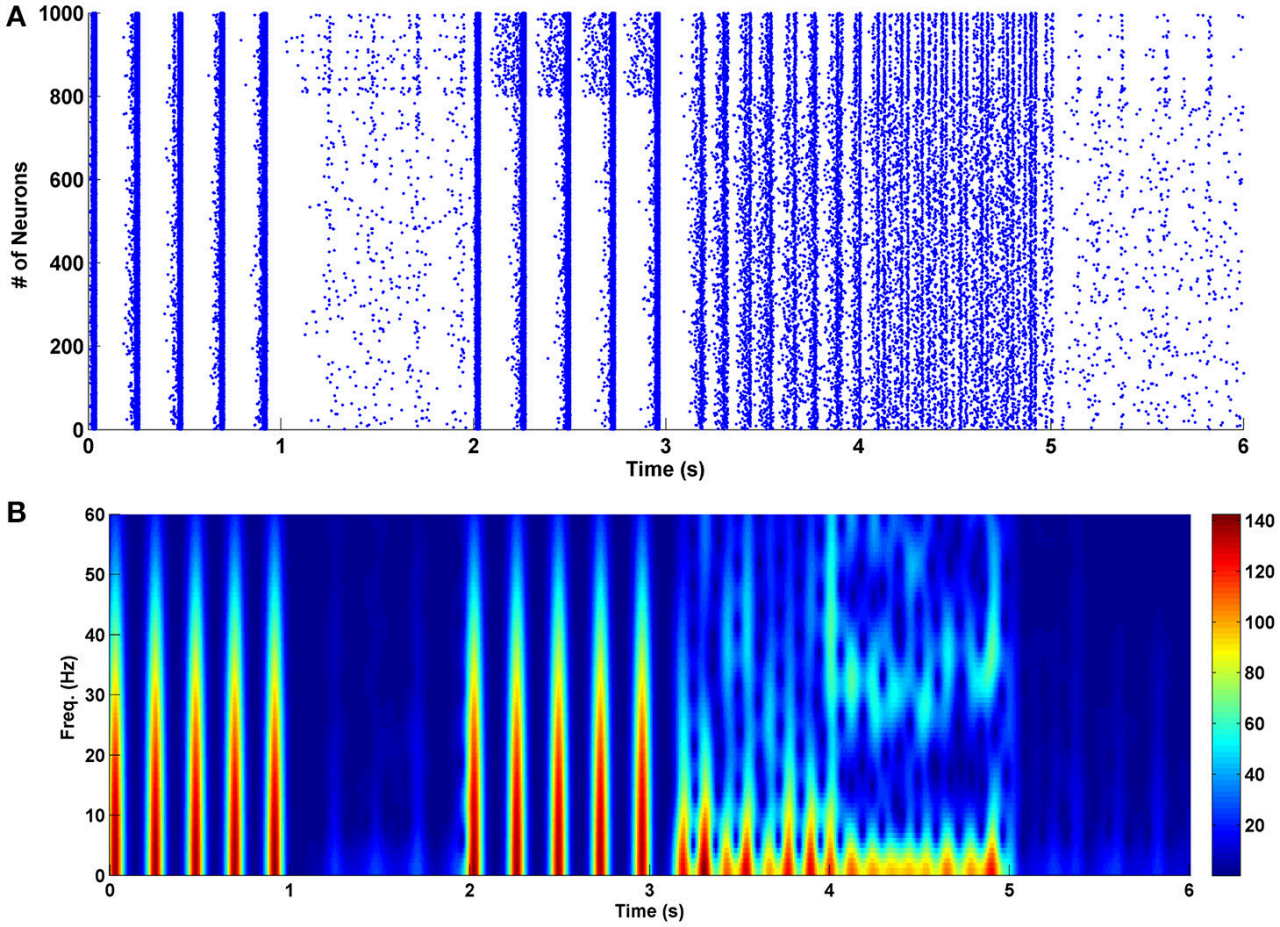
The effect of changes in various parameters of the network on its performance in the chronic mode. **(A)** The raster plot, and **(B)** the spectrogram of PSCs. Between 0 and 1 s the network is in the chronic mode, from 1 to 2 s the strength of the inputs of the excitatory neurons are reduced by 24% of their normal values, from 2 to 3 s the input strength of interneurons is increased by 100%, from 3 to 4 s the excitatory connections are weakened by 6%, between 4 and 5 s strength of the inhibitory connections are augmented by 40% and between 5 and 6 s activity of the astrocyte transporters are enhanced by 37.5%.

To prevent the formation of drug-related memories in the chronic opioid consumption, we manipulated some key parameters of the proposed model. This is demonstrated in Figure 4. The chronic mode is given between 0 and 1 s. Between 1 and 2 s, the amplitude of excitatory neuron's input is decreased such that the synchronization disappears. It was observed that when the stimulus signal reaches the value of *K*_*Exc*_ = 3.8, the synchronization is completely disappeared (24% decrease compared to the normal mode). As it can be seen decreasing the amplitude of the excitatory stimulation signal can disrupt synchronized patterns in the theta frequency. Since the excitatory signals are driven by presynaptic neurons, therefore, it seems that presynaptic excitatory outputs (such as glutamate density) have significant roles in pathological memory formation process. So, decreasing the synaptic glutamate can prevent the formation of pathological drug-related memories. Between 2 and 3 s, the amplitude of the interneuron's input is increased by 100% (*K*_*Inh*_ = 4). It can be seen that despite the large changes in the input, synchronized activities still exist. Therefore, we can conclude that increasing the interneuron's input (increasing the inhibition caused by the interneurons) cannot prevent the formation of drug-related memories.

Next, between 3 and 4 s, the excitatory synaptic connections are weakened such that the synchronization is died out. It was observed that when the attenuation is 6% less than the normal value (*s*_*Exc*_ = 0.47), the synchronous pattern is disappeared. Since the strength of the excitatory synapses is correlated to the conductance of the NMDA and AMPA receptors, therefore, reducing the conductance of these receptors may prevent pathological memory formation process during chronic opioid consumption. The same idea (which was predicted by the proposed model) was used previously by some researchers in experiment to prevent the formation of the addiction memories (Mao, 1999; Herman et al., 2003; Zweifel et al., 2008; van Huijstee and Mansvelder, 2015; Vengeliene et al., 2015).

In the next step, between 4 and 5 s we increased the strength of the inhibitory synaptic connections to eliminate synchronization in the firing pattern of neurons. Our results indicate that when the strength of the inhibitory network is amplified by 40% (*s*_*Inh*_ = −1.4), the firing patterns become the same as in the acute state. In fact, low-frequency synchronized patterns are changed to the high-frequency synchronized patterns. More increase in the strength of inhibitory synaptic connections can completely block the synchronization.

Between 5 and 6 s, the activity of astrocytic transporters is increased by 37.5% (δ = 0.5). In this case, the synchronous oscillations are completely blocked. Also, there is evidence that astrocytic glutamate transporters -which clear the synapse from the glutamate- are involved in the morphine dependency (Wang et al., 2017). Therefore, based on the simulation results and Wang et al.'s observation (Wang et al., 2017), we suggest that one way to prevent the formation of pathological memories can be the stimulation of the astrocytic glutamatergic transporters to clear the synapse from the extra glutamates.

**Figure 5:**
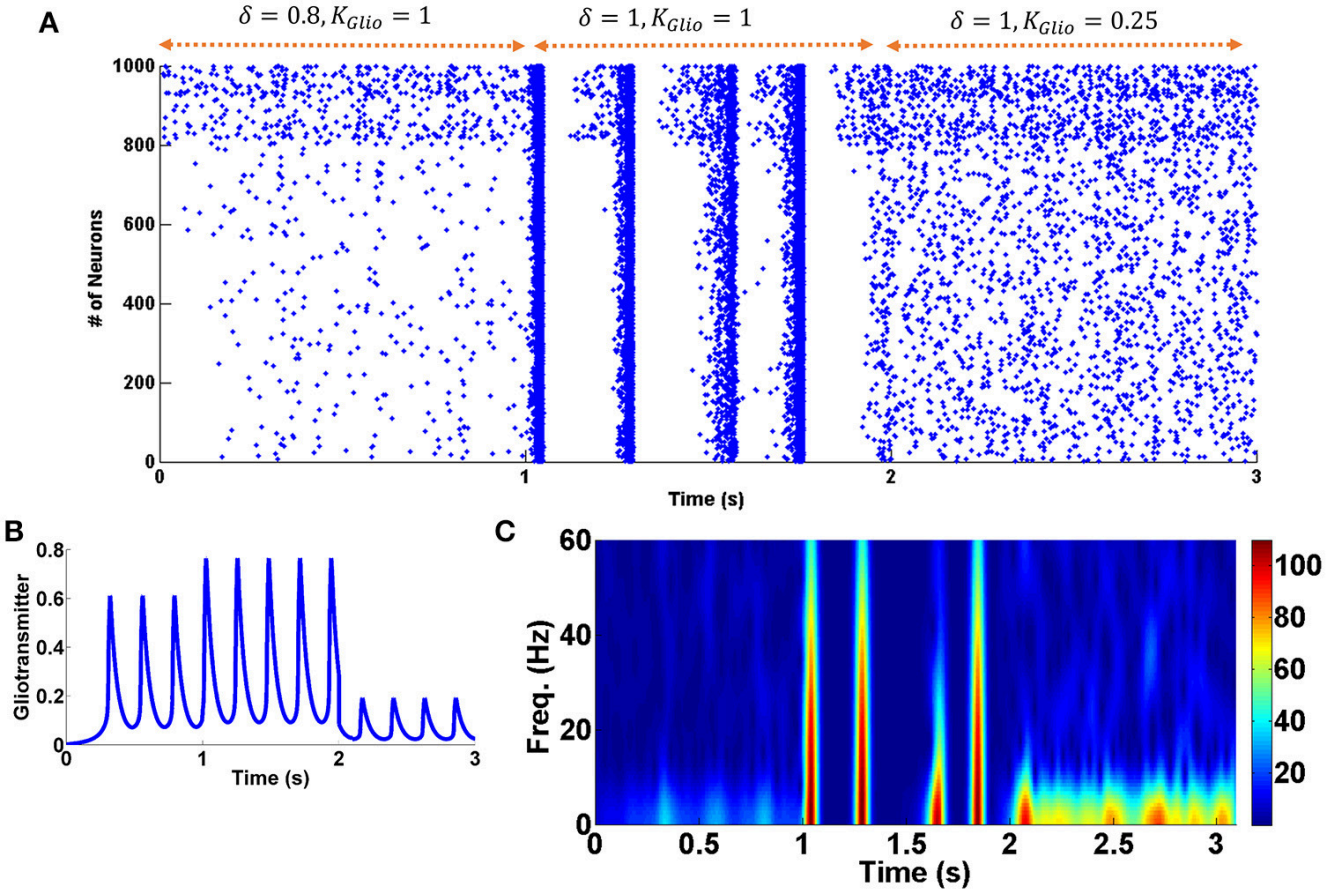
Changes in the activity of astrocytic transporters and gliotransmitter release. **(A)** Raster plot. Between 0 and 1 s withdrawal state is displayed. From 1 to 2 s, astrocyte transporters are inactive. From 2 to 3 s, while the astrocytic transporters are still inactive, the gliotransmitter release has been decreased. **(B)** Gliotransmitter dynamics. **(C)** Shows the spectrogram of PSCs.

As shown in Figure 2, during withdrawal (between 6 and 8 s), the firing rate of the neurons decreases significantly and the patterns of firing are random. Significant reduction in the firing rate of the neurons has been reported in some other researches during withdrawal period (Diana et al., 1995, 1999; Enrico et al., 2016; Meye et al., 2017). Furthermore, it has been suggested that changes in excitatory and inhibitory inputs are responsible for the reduction of the firing rate of the neurons at this stage (García-Pérez et al., 2015; Enrico et al., 2016). Besides that, according to the empirical evidence during withdrawal period the activity of the astrocytic transporters is significantly reduced (Scofield and Kalivas, 2014). Therefore, in the withdrawal mode, the activity of the astrocytic transporters is significantly reduced in the model (δ = 1). This case is shown between 1 and 2 s. It can be seen that by disabling the astrocytic transporters, the firing pattern of the neurons is synchronized and burst-type APs are observed. The similarity of this case with the chronic mode can suggest that the previously formed pathological memory is recalled. As a result, the likelihood of relapse is very high. By changing some of the parameters, it was observed that if the gliotransmitter release is weakened, synchronized fluctuations (that could indicate the relapse) are eliminated. This case is shown between 2 and 3 s in Figure 5.

## Conclusion

Overall, in this paper, it has been shown that opioid consumption, by applying the disinhibitory effect can change firing patterns of the neurons. It has been demonstrated that during chronic opioid consumption neurons are synchronized and fire in the theta frequency range with the burst patterns. Since based on experimental observations induction of the theta frequency is linked to the memory formation process, therefore, probably our results suggest that the neurons learn the memory of addiction process. Efficient methods to prevent the formation of the pathological memory are (1) increasing the input of the excitatory neuron, (2) weakening (or decreasing) the excitatory synaptic connections, or (3) stimulation of the astrocytic transporters. By reducing the activity of the pre-synaptic neurons, the excitatory neurotransmitters can be reduced. Attenuation of the excitatory synaptic connections is possible by decreasing the conductance of the related receptors such as NMDARs and AMPARs in glutamatergic synapses. Stimulation of astrocytic transporters is also a pragmatic and realistic approach (Sweeney et al., 2017). During withdrawal period firing of the neurons diminished significantly with random-like patterns. The occurrence of relapse is probably due to reduced activity of astrocytic transporters. Based on simulation results, increased activity of astrocytic transporters, or reduction of gliotransmitter release, can prevent the retrieval of pathologic memory during withdrawal period. Generally, these findings may unveil new aspects of the opioid-induced memories and relapse.

## Author contributions

MB, FB, and MJ conceived the work. MB developed the codes and performed the computations. MB, FB, and MJ analyzed the results. MB and FB wrote the manuscript.

### Conflict of interest statement

The authors declare that the research was conducted in the absence of any commercial or financial relationships that could be construed as a potential conflict of interest.
